# Psychometric properties of the Italian Place Standard Tool for Children: validation in primary school children within the PNC Clima national programme

**DOI:** 10.3389/fpubh.2026.1947358

**Published:** 2026-09-21

**Authors:** Alessandra Pandolfo, Andrea Perri, Giustino Carlino, Pietro Alfano, Silvia Torta, Angela Marina Montalbano, Giusy Daniela Albano, Anna Bonomolo, Vito Michele Rosario Muggeo, Stefania La Grutta, Velia Malizia

**Affiliations:** 1 Institute of Translational Pharmacology (IFT), National Research Council (CNR), Palermo, Italy; 2 Department of Economics, Business and Statistics (dSEAS), University of Palermo (UNIPA), Palermo, Italy; 3 Department of Earth and Marine Sciences (DiSTeM), University of Palermo (UNIPA), Palermo, Italy; 4 Department of Culture and Society, University of Palermo (UNIPA), Palermo, Italy

**Keywords:** children, item response theory, Place Standard Tool, polychoric factor analysis, semantic equivalence, validation

## Abstract

**Background:**

The Place Standard Tool Children’s Version (PST-C) lets children evaluate the quality of places where they live. Developed in Scotland and adapted in over a dozen countries, it had no validated Italian version. This study evaluated the psychometric properties of the Italian PST-C within a case study of the national research programme PNC E.1 “Salute, Ambiente, Biodiversità e Clima” (PNC Clima).

**Methods:**

A sample of 131 children attending the last 2 years of primary school and the first 2 years of middle school (69 boys, 62 girls, aged 9 to 12 years) completed the 14-item instrument on a three-point ordinal scale. Psychometric evaluation included floor and ceiling effects, internal consistency (Cronbach’s α and McDonald’s ω), structural validity from polychoric correlations, item functioning under Samejima’s graded response model, known-groups validity. Translation equivalence was evaluated using both conventional expert review procedures and multilingual sentence embeddings.

**Results:**

Internal consistency was good: α = 0.83 (95% CI [0.77, 0.87]) and ω = 0.86 (95% CI [0.82, 0.89]). Parallel analysis and confirmatory factor analysis (CFI = 0.904, RMSEA = 0.058) supported a unidimensional structure, with all polychoric loadings ≥ 0.42. Semantic equivalence between versions was high (mean cosine similarity = 0.86). Two findings warrant caution: all items showed ceiling effects above the 15% threshold, and no known-group differences survived correction for multiple testing.

**Conclusion:**

Combining COSMIN-based validation, item response theory and AI-based semantic equivalence, the Italian PST-C provides a reliable measure of perceived place quality in children. Future studies should evaluate an expanded response scale and confirm these findings in larger, more diverse populations.

## Introduction

The quality of the everyday environments in which children grow up shapes their health, autonomy and wellbeing (1–3). Children develop meaningful relationship with the places and natural environment in which they live, and these connections support coping and development (4). Article 12 of the United Nations Convention on the Rights of the Child also establishes children’s right to express views on matters that affect them and to have those views taken seriously. Initiatives such as the Child Friendly Cities programme translate this principle into local planning practice (5).

What is known about the environmental features that matter to children comes from public health, environmental psychology and urban planning? On the physical side, green and natural space, safe and attractive places to play, walkable and cyclable streets, low exposure to motorised traffic and the upkeep of public space have all been associated with children’s physical activity, independent mobility, cognitive and socio-emotional development and mental wellbeing (1, 2). The social side matters as much. Perceived safety, neighbourhood cohesion, opportunities for informal contact with peers and adults, and the sense of being listened to determine whether children actually use the spaces available to them, and they are among the strongest correlates of independent mobility and outdoor play (3, 6). The two interact: a well-equipped playground that children do not perceive as safe, or a green area that adults do not allow them to reach unaccompanied, contributes little to their daily experience. Most of this evidence, however, rests on objective or adult-reported measures. Children’s own appraisal of their neighbourhood is documented far less systematically (2, 7).

Direct measurement of children’s perceptions has usually relied on youth adaptations of adult questionnaires, such as the Neighborhood Environment Walkability Scale for Youth (8), or on item sets built for a single study and reported without formal psychometric evaluation. Participatory and mapping-based approaches, including softGIS methods in which children locate and rate meaningful places in their neighbourhood, have produced rich context-specific evidence (6, 9). They are also demanding to administer at scale, and the data they yield are hard to compare across settings. Two gaps run through this literature. Instruments are rarely validated with children of primary-school age, and they rarely cover physical and social qualities of place within one framework. A third problem is specific to adaptation: when an instrument is translated, semantic equivalence between source and target versions is usually assumed rather than demonstrated. Urban planners and public health professionals therefore require instruments that let children evaluate the places where they live in a structured, age-appropriate and psychometrically defensible way (5, 7, 8).

The Place Standard Tool (PST), developed in Scotland by the Scottish Government, NHS Health Scotland, and Architecture and Design Scotland (10), is one of the most widely adopted frameworks for the participatory assessment of place quality. It organizes the physical and social dimensions of a place into fourteen domains, ranging from movement and transport to safety, identity and influence, and asks respondents to rate each one. The tool has been adopted well beyond Scotland. Published applications include a national internet survey in Cyprus (11); a Spanish adaptation whose psychometric properties were evaluated in a municipality in Valencia and subsequently applied to compare urban and rural settings (12, 13); an implementation in North Macedonia, where the Macedonian version demonstrated good internal consistency (Cronbach’s α of 0.89) (14); and an analysis of more than 8,000 responses from northern England examining the tool’s capacity to capture place-related inequalities (15). A version specifically designed for children of approximately 6 to 12 years, featuring simplified wording, illustrated prompts and a reduced three-point response format, was released in 2022 (16); a distinct version for young people aged 12 and over, which retains a seven-point response scale, was released alongside it (16). Despite its widespread adoption, the Place Standard Tool has primarily been used as a participatory community engagement tool rather than as a psychometrically validated measurement instrument, and evidence regarding its measurement properties remains limited (11, 15). For the Children’s Version (PST-C) such evidence is particularly scarce, and no validated Italian version has yet been published.

The present validation was conducted within an objective case study of PNC Clima, the programme E.1 “Salute, Ambiente, Biodiversità e Clima” of the Italian National Plan for Complementary Investments to the National Recovery and Resilience Plan. The programme supports applied research on the relationships between climate, environment and health within a One Health perspective. One of its case studies focuses on how children perceive and evaluate their everyday urban environments in the context of climate change. The school-based administration of the PST-C reported here forms part of that case study, and the availability of a validated Italian version is a prerequisite for the programme’s subsequent monitoring activities.

Validating the PST-C in Italian raises two methodological problems. The first concerns the response format: items are ordinal and include only three response categories. Under these conditions, Pearson correlation-based factor analysis tends to underestimate inter-item associations; therefore, polychoric correlations provide a more appropriate basis for structural analyses (17, 18). The second challenge relates to translation quality, which is typically assessed through qualitative procedures involving independent forward translation and expert reconciliation (19). Recent advances in multilingual sentence embeddings (20, 21) offer a quantitative complement to these procedures, allowing semantic similarity between the source and target versions of each item to be computed directly in a shared vector space.

Following the COSMIN methodological standards for evaluating measurement properties (22, 23), we examined: (a) item functioning, including floor and ceiling effects; (b) internal consistency; (c) structural validity, through parallel analysis, exploratory and confirmatory factor analysis on polychoric correlations, and Samejima’s graded response model (24); (d) known-groups validity across sex, school and grade; and (e) the semantic equivalence of the Italian translation.

## Materials and methods

### Study context, participants and procedure

Data were collected in the schools participating in the PNC Clima case study on children’s perception of urban environments. Participants were 131 primary school children (69 boys, 52.7%; 62 girls, 47.3%) from four school buildings belonging to four different state comprehensive institutes (istituti comprensivi statali), attending grades 4 and 5 of Italian Primary School and grades 1 and 2 of Italian Middle School, age range of 9 to 12 years. The questionnaire was administered in paper-based format during regular school hours. No private or state-subsidised (paritarie) schools were involved. The four institutes are all located in the municipality of Palermo, Sicily, and serve different districts of the same urban area. Participation was voluntary. Written informed consent was obtained from the parents or legal guardians of every child before administration. A member of the research team then introduced the study to each class in age-appropriate language, explaining what it was about, what the children would be asked to do and how long it would take. The children were told that there were no right or wrong answers, that their answers would be used only for research and reported only in aggregate form, and that their teachers would not see individual responses. They were invited to ask questions, and verbal assent was obtained individually from each child before the questionnaire was distributed. We also made clear that they could decline to take part, leave any question unanswered, or stop at any moment, without giving a reason and without consequence. Some did stop before reaching the end, which accounts for part of the item-level missing data reported below. These procedures follow the principles of the International Charter for Ethical Research Involving Children (25). The study was approved by the Ethics Committee of *Comitato Etico Locale Palermo 1* (Approval No. 25, date: 30/10/2024). Item-level missing data ranged from 1.5 to 9.2%. The listwise-complete dataset included 98 children for analyses requiring complete responses on all 14 items and 121 children for the confirmatory factor analysis.

### Instrument

The instrument used here is the Children’s Version of the Place Standard Tool (PST-C), released in 2022 for children of roughly 6 to 12 years (16). It is not the same as the Young People’s Version, which is intended for respondents aged 12 and over and keeps the seven-point response scale of the adult tool (16). The version translated and validated in this study is the Children’s Version, with its three-point scale. The PST-C asks children to evaluate fourteen domains of their living environment: streets and active movement, natural spaces, places for play, walking and cycling, public transport, traffic and parking, feeling heard, care and cleanliness of public space, perceived safety, identity and pride, services and amenities, work and the local economy, housing and neighbours, and social contact. The questionnaire contains one scored question per domain, so there are fourteen closed items in total, no sub-items, and a total score ranging from 14 to 42. Each domain is rated on a three-point scale. The verbal anchors in the source tool are 1 = “not good, lots of improvement needed,” 2 = “okay, some improvement needed” and 3 = “great, it’s good or as good as it can be.” Each domain also carries two open-ended prompts, for twenty-eight open questions overall. The first is What is good now? The second appears in the source tool as What would you use your magic wand on? and was rendered in the Italian version as Cosa si potrebbe migliorare? (What could be improved?), a wording judged more transparent for classroom administration during the reconciliation step. Illustrated examples and supplementary probing questions accompany each domain; they support reflection and are not scored. Table 1 gives, for each of the fourteen domains, the domain heading and the scored question as worded in the English source tool. For access to the full original scale and the accompanying facilitator guidance, please refer to reference (16). Translation and cross-cultural adaptation

**Table 1:** The fourteen domains of the children’s version of the place standard tool, with the domain heading and the scored question as worded in the English source tool (16).

Item label	PST-C domain (source heading)	Scored question (English source wording)
Streets and movement	Streets, squares and buildings	What are the streets, squares and buildings like in my place?
Natural spaces	Nature—parks, trees, animals, plants	How much nature is there around my place and is it easy to get to?
Places for play	Places to play	What spaces are there for me to play and have fun in and around my place?
Walking and cycling	Walking, wheeling and cycling	How easy is it to walk, scoot, wheel or cycle to places you need and want to go?
Public transport	Buses, trains, trams, boats	Is it easy for me to get where I need to go to by bus, train (or other public transport)?
Traffic and parking	Traffic and parking	How does traffic in my place affect me when I’m out and about?
Feeling heard	Having our say and being listened to	When things happen in my place do people include me in decisions and listen to what I say?
Care and cleanliness	Fixed, clean and looking nice	Is my place clean and are things well cared for?
Feeling safe	Feeling safe	Do I feel safe in my place?
Identity and pride	Feeling proud and part of my place	Do I feel proud and a part of my place?
Services and amenities	Schools, libraries, shops and other services	What are the schools, libraries, shops and places to get what I need like?
Work and economy	Jobs and places to work	Are there places for people to work and get a good job in my place?
Housing and neighbours	Homes, friends and neighbours	How well do the homes in my place meet my needs and my family’s needs?
Social contact	Meeting and talking with people	How nice are the nearby places where I meet with other children and trusted adults?

Each domain contributes one rating on a three-point scale and two open-ended prompts. Short item labels used in the remaining tables and figures are given in the first column.

The validation followed established procedures for the translation and cross-cultural adaptation of measurement instruments (19). Two independent bilingual experts performed forward translations of the English source items, adapting wording and examples to the Italian primary-school context. A third independent expert compared the two translated versions, resolved discrepancies and verified that each reconciled item remained conceptually and semantically equivalent to the original while remaining understandable to young children. The reconciled version was subsequently pilot-tested for comprehensibility with a group of 10 children of the target age range before administration. No expert committee was convened beyond the three bilingual experts described above.

To complement the qualitative cross-cultural adaptation process, we conducted an objective quantitative assessment of semantic equivalence. This last analysis was carried out by the study authors, and not by the bilingual experts, whose involvement was limited to the translation and reconciliation steps. The English and Italian wording of each item was encoded using the multilingual sentence-embedding model *paraphrase-multilingual-MiniLM-L12-v2* (20, 21), which maps sentences from more than 50 languages into a shared 384-dimensional vector space. For each item we computed the cosine similarity between the English source and its Italian translation. As a benchmark, we also calculated the mean similarity between each Italian item and the English source items of the other thirteen domains. A faithful translation would be expected to show substantially greater similarity to its own source than to conceptually unrelated items.

### Statistical analysis

Analyses were conducted in Python 3.11 using the packages *factor_analyzer* (0.5.1 or later), *pingouin, semopy, girth* and *statsmodels*. Given the three-category ordinal response format, analyses were based primarily on polychoric correlation matrix (estimated by pairwise maximum likelihood) wherever appropriate instead of Pearson correlations, which tend to underestimate associations among coarsely categorized ordinal variables (17, 18). The analytic flow followed the COSMIN standards for evaluating measurement properties (22, 23). Descriptive statistics included floor and ceiling effects using the conventional 15% threshold. Internal consistency was estimated with Cronbach’s α and McDonald’s total ω; ω is generally preferred when items are congeneric rather than tau-equivalent (26, 27). Ninety-five percent confidence intervals were estimated using non-parametric bootstrap with 1,000 resamples (28). Factorability was assessed using the Kaiser-Meyer-Olkin index and Bartlett’s test of sphericity (29). The number of factors was determined primarily through parallel analysis based on 500 randomly generated Gaussian datasets, one of the most accurate factor-retention methods under realistic conditions (30). The eigenvalue-greater-than-one criterion was used only as a secondary reference, because of its tendency to over extract factors (31). Exploratory factor analysis (EFA) was performed using minimum residual extraction with oblique Promax rotation, and was fitted to both the polychoric and the Pearson matrices for comparison (29). A congeneric one-factor confirmatory factor analysis (CFA) model was estimated using *semopy* under maximum likelihood and evaluated using conventional goodness-of-fit indices, while acknowledging the known limitations of these indices when applied to ordered categorical data (32). Samejima’s graded response model (GRM) was estimated using marginal maximum likelihood to obtain discrimination and threshold parameters (24, 33). Known-groups validity was examined across sex (Mann–Whitney U), school and grade level (Kruskal-Wallis) with Holm correction applied for multiple testing across the 14 questionnaire domains.

The reference values used to interpret each measurement property were set in advance and applied throughout (22, 23, 27, 29, 32, 33). Floor and ceiling effects were treated as problematic above 15% of respondents in an extreme category. Internal consistency was read as adequate for group-level use from α and ω ≥ 0.70 and as good from ≥ 0.80. Corrected item-total correlations had to exceed 0.30, and values between 0.30 and 0.40 were flagged as weak. Sampling adequacy was acceptable at KMO ≥ 0.60 and good at KMO ≥ 0.80, and factor loadings were treated as salient from 0.40. Model fit was mixed but broadly supportive of the one-factor solution: CFI met the prespecified criterion and RMSEA was acceptable, whereas TLI fell slightly below the 0.90 threshold. Graded response model discrimination parameters were read as low below 1.0, moderate between 1.0 and 1.7, and high above 1.7. For the semantic equivalence analysis, cosine similarities of 0.80 or above between matched pairs were taken to indicate close correspondence between source and translation. Statistical significance was set at a two-sided α of 0.05, with Holm correction across the 14 domain-level comparisons; because the known-groups analysis was exploratory, the direction and size of the differences were considered alongside the corrected *p* values.

## Results

### Sample and item descriptive statistics

Table 2 summarizes the sample and Table 3 presents the item-level descriptive statistics. Item means ranged from 2.05 (*Traffic and parking*) to 2.36 (*Housing and neighbours*). The median response was 2 for all item, and skewness was mildly negative throughout (between −0.48 and −0.05), indicating a general tendency for children to evaluate their living environment positively. All 14 items exceeded the conventional 15% ceiling-effect threshold, with ceiling rates ranging from 24.0 to 44.0%. Four items showed ceiling effects exceeding 40%: *Identity and pride* (44.0%) *Housing and neighbours* (43.1%) *Services and amenities* (42.9%) *Natural spaces* (40.6%). Floor effects were less pronounced (7.3 to 24.8%) and exceeded the threshold for five items (Walking and cycling, Public transport, Traffic and parking, Feeling heard, Care and cleanliness), with the highest value observed for Traffic and parking (24.8%). Figure 1 illustrates both floor and ceiling effects.

**Table 2:** Characteristics of the validation sample (*N* = 131 primary school children): sex, school grade and school of recruitment, with absolute frequencies and percentages.

Variable	*n*	%
Sex: female	62	47.3
Sex: male	69	52.7
Grade 4—elementary school	20	15.3
Grade 5—elementary school	19	14.5
Grade 1—middle school	54	41.2
Grade 2—middle school	38	29.0
School 1	39	29.8
School 2	20	15.3
School 3	55	42.0
School 4	17	13.0

No third-grade classes were sampled.

**Table 3:** Descriptive statistics for the 14 PST-C items: number of valid responses (N), mean (M) and standard deviation (SD) on the 1–3 response scale, and percentage of children at the lowest (floor) and highest (ceiling) response category.

Item	Domain	N	M	SD	Floor %	Ceiling %
Streets and movement	Streets and active movement	129	2.10	0.61	14.0	24.0
Natural spaces	Natural spaces	128	2.26	0.70	14.8	40.6
Places for play	Places for play	125	2.22	0.65	12.8	34.4
Walking and cycling	Walking and cycling	129	2.22	0.71	16.3	38.0
Public transport	Public transport	126	2.14	0.72	19.8	34.1
Traffic and parking	Traffic and parking	125	2.05	0.74	24.8	29.6
Feeling heard	Feeling heard	124	2.15	0.73	20.2	34.7
Care and cleanliness	Care and cleanliness	119	2.06	0.69	21.0	26.9
Feeling safe	Perceived safety	121	2.23	0.69	14.9	38.0
Identity and pride	Identity and pride	125	2.34	0.65	9.6	44.0
Services and amenities	Services and amenities	126	2.33	0.66	10.3	42.9
Work and economy	Work and local economy	122	2.26	0.59	7.4	33.6
Housing and neighbours	Housing and neighbours	123	2.36	0.62	7.3	43.1
Social contact	Social contact and meeting	125	2.24	0.66	12.8	36.8

Item labels are the Italian short names; the corresponding domain is given in English.

**Figure 1:**
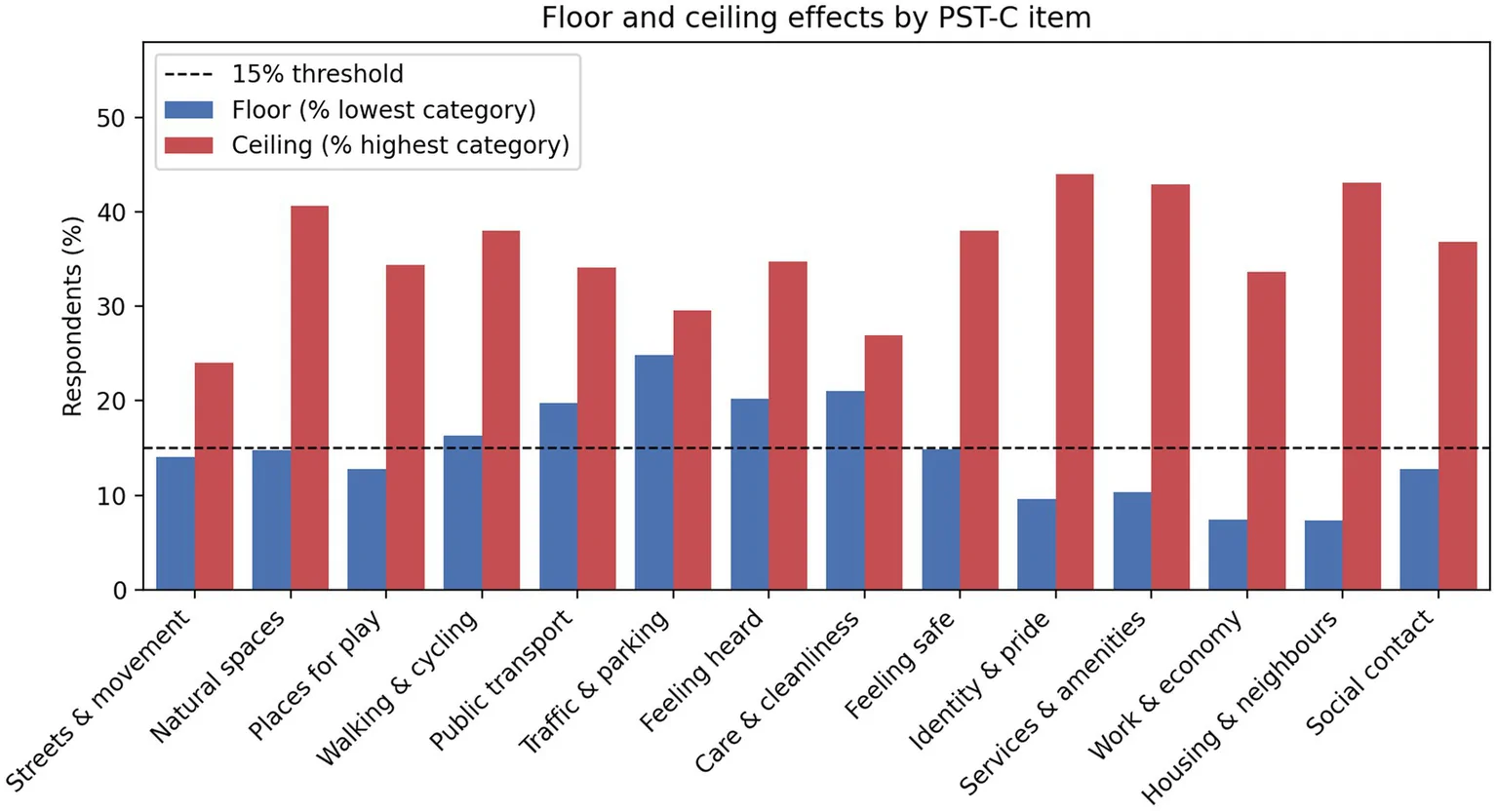
Floor and ceiling effects for the 14 PST-C items, shown as the percentage of children choosing the lowest (floor, blue bars) and highest (ceiling, red bars) response category. The dashed line marks the conventional 15% criterion.

### Internal consistency

Internal consistency was good: Cronbach’s *α* = 0.829 (95% bootstrap CI [0.769, 0.870]) and McDonald’s ω = 0.863 (95% bootstrap CI [0.824, 0.893]), both exceeding the 0.80 thresholds commonly recommended for applied use (27, 34). Corrected item-total correlations ranged from 0.33 to 0.59 (Table 4), all above the conventional minimum criterion of 0.30. Four items fell below the more stringent 0.40 criterion: *Feeling heard* (0.33), *Identity and pride* (0.34), *Services and amenities* (0.35) and *Housing and neighbours* (0.39). Deleting any individual item would leave α essentially unchanged (0.81 to 0.83); therefore, no item removal was justified on reliability grounds. All corrected item-total correlations thus exceeded the conventional 0.30 minimum, and the four values below 0.40 identify the weaker items noted above.

**Table 4:** Item-level reliability indices (*N* = 98 listwise-complete cases): corrected item-total correlation, computed between each item and the sum of the remaining 13 items, and the value Cronbach’s α would take if the item were deleted.

Item	r item-total (corrected)	α if item deleted
Streets and movement	0.50	0.82
Natural spaces	0.45	0.82
Places for play	0.54	0.81
Walking and cycling	0.55	0.81
Public transport	0.46	0.82
Traffic and parking	0.51	0.81
Feeling heard	0.33	0.83
Care and cleanliness	0.59	0.81
Feeling safe	0.57	0.81
Identity and pride	0.34	0.82
Services and amenities	0.35	0.83
Work and economy	0.41	0.82
Housing and neighbours	0.39	0.82
Social contact	0.46	0.82

### Polychoric correlations and factorability

Polychoric correlations among the 14 items ranged from −0.01 to 0.58 (mean 0.33) and were consistently positive (Figure 2). This pattern already suggests the presence of a dominant common dimension. Sampling adequacy was good (KMO = 0.814), all item-level KMO values ≥0.71 (29) and Bartlett’s test of sphericity was significant, *χ*^2^ = 333.6, *p* < 0.001, indicating the data were suitable for factor analysis.

**Figure 2:**
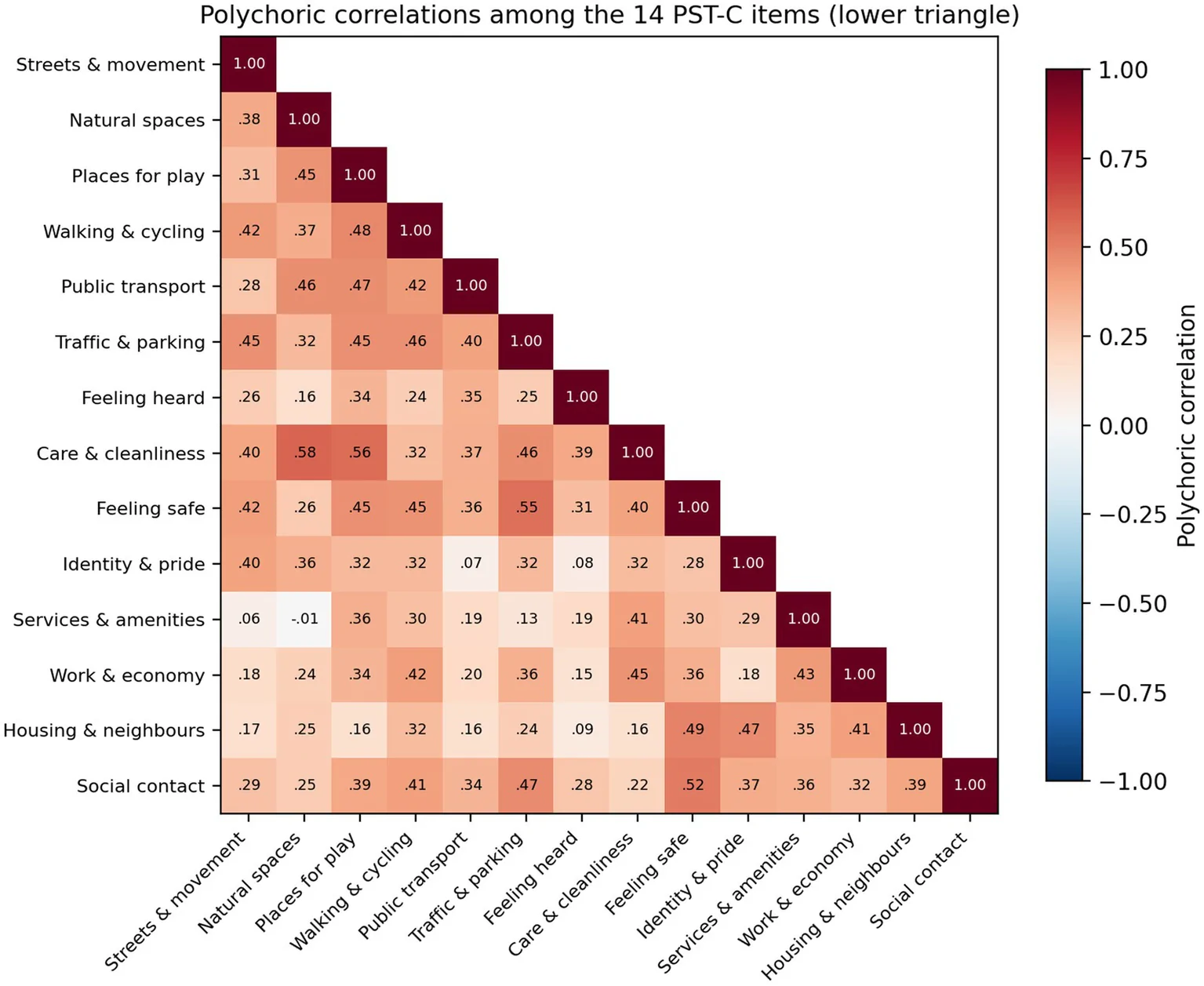
Polychoric correlation matrix among the 14 PST-C items (lower triangle shown). Correlations range from −0.01 to 0.58 with a mean of 0.33 and are almost uniformly positive.

### Dimensionality

Parallel analysis and inspection of the scree plot supported a unidimensional solution (Figure 3). The first observed eigenvalue was 5.37 on the polychoric matrix (4.43 on the Pearson matrix), substantially exceeding the 95th percentile of eigenvalues obtained from random data (1.88). The second observed eigenvalue (1.46 and 1.44 respectively) already fell below the corresponding random-data threshold (1.64). Although the Kaiser criterion suggested retaining three factors, this method is known to overestimate dimensionality (31). Accordingly, greater weitht was given of the results of the parallel analysis (30).

**Figure 3:**
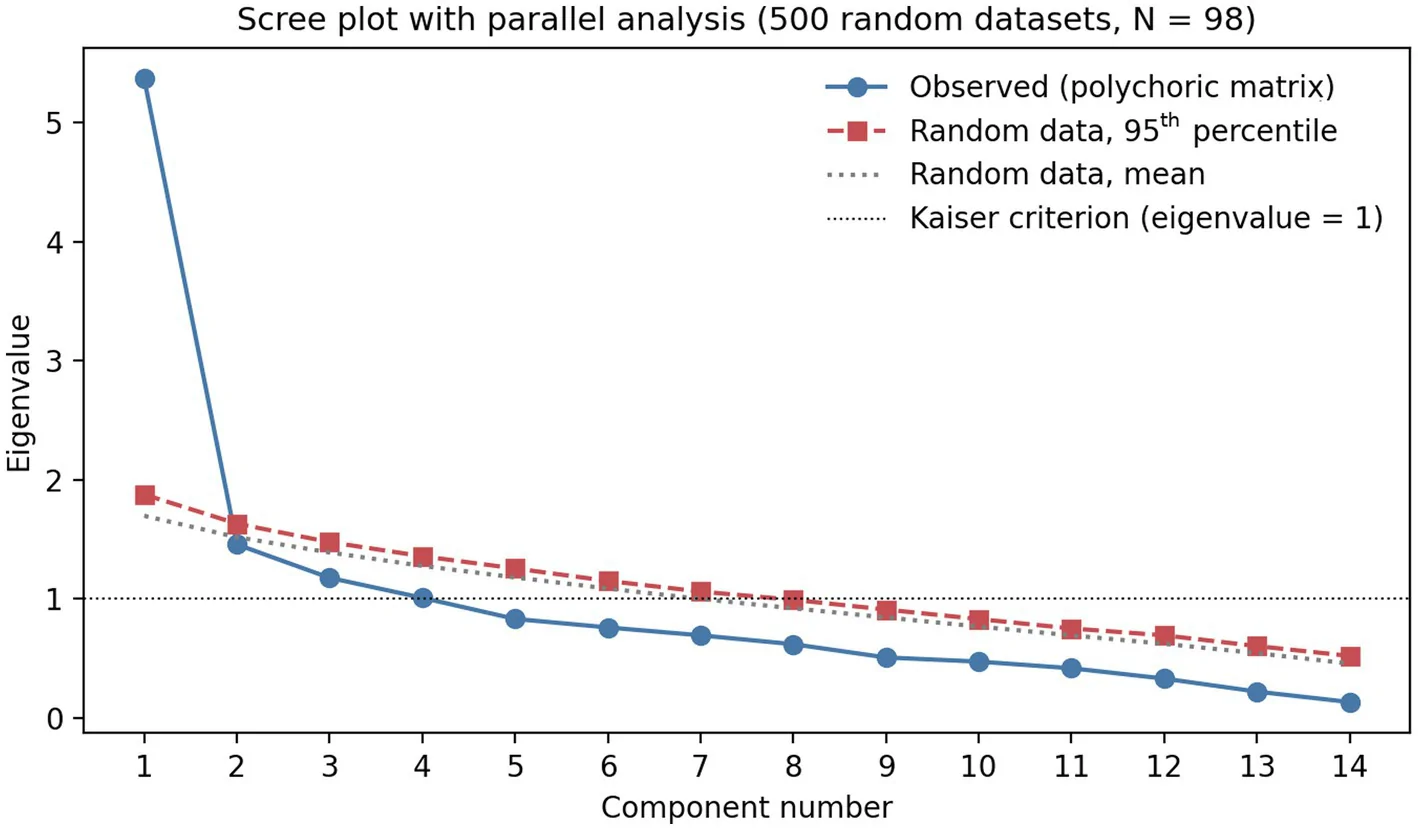
Scree plot with parallel analysis on the polychoric correlation matrix. Observed eigenvalues (blue circles) are compared with the mean (grey dotted line) and the 95th percentile (red squares) of eigenvalues from 500 random Gaussian datasets of the same size (*N* = 98, 14 variables). The dotted line at eigenvalue = 1 marks the Kaiser criterion.

### Exploratory factor analysis

The one-factor solution based on the polychoric matrix explained 33.8% of the variance. All factor loadings were positive and exceeded the conventional 0.40 benchmark (29), ranging from 0.42 (Feeling heard) to 0.70 (Feeling safe) (Table 5; Figure 4). Factor loadings estimated from the Pearson matrix were on average 0.067 lower in absolute value, as expected when analyzing ordinal items with only three responses categories (18).

**Table 5:** Standardized loadings of the one-factor EFA solution (minimum residual extraction, promax rotation), estimated on the polychoric and on the pearson correlation matrix.

Item	λ (polychoric)	λ (Pearson)
Streets and movement	0.55	0.51
Natural spaces	0.55	0.48
Places for play	0.69	0.62
Walking and cycling	0.67	0.60
Public transport	0.55	0.50
Traffic and parking	0.67	0.59
Feeling heard	0.42	0.36
Care and cleanliness	0.67	0.64
Feeling safe	0.70	0.63
Identity and pride	0.50	0.40
Services and amenities	0.44	0.39
Work and economy	0.54	0.47
Housing and neighbours	0.48	0.40
Social contact	0.61	0.50

Polychoric loadings are the reference values; Pearson loadings are reported as a robustness check.

**Figure 4:**
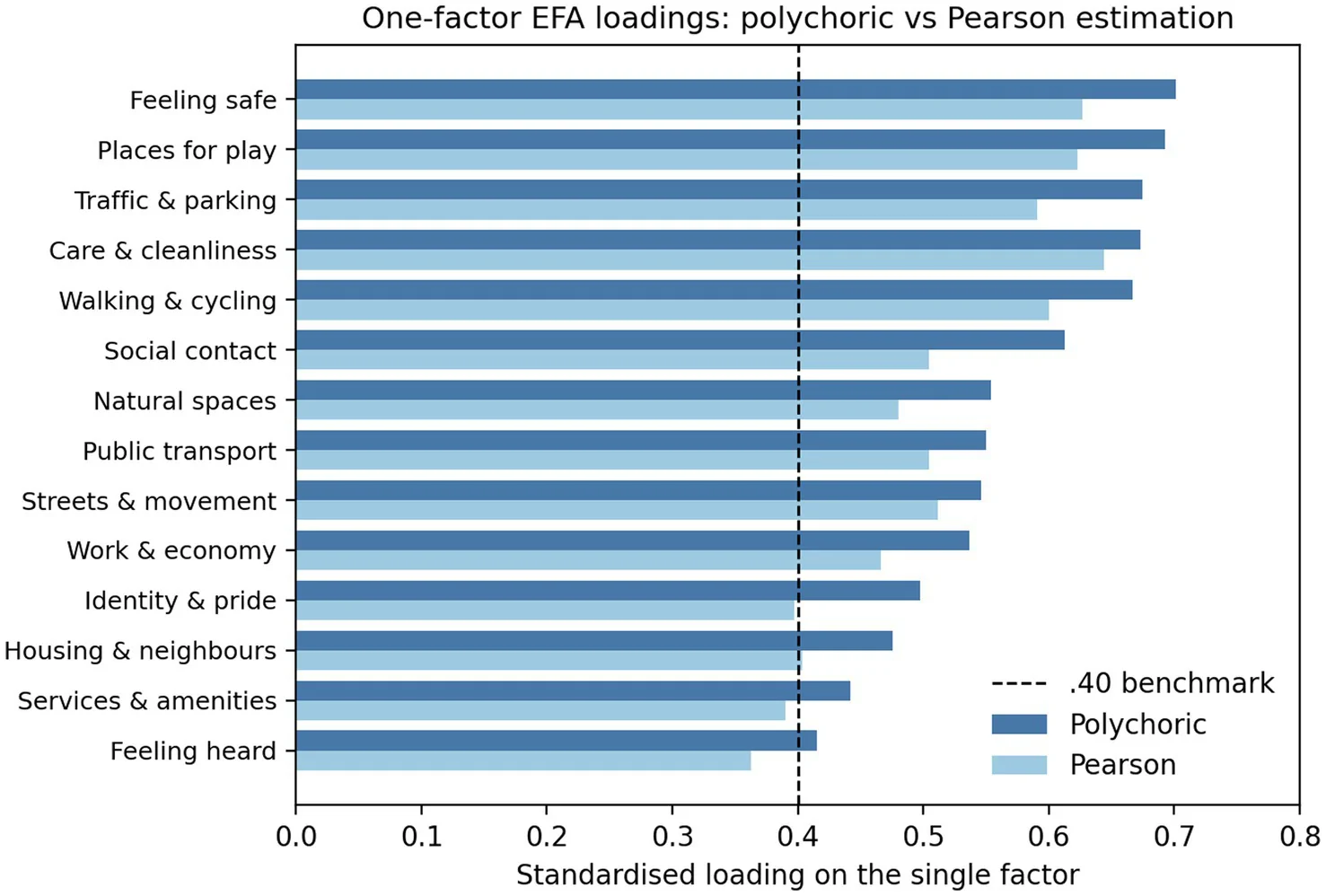
Standardized loadings of the one-factor EFA solution, sorted by size, estimated on the polychoric (dark blue) and Pearson (light blue) correlation matrices. The dashed line marks the 0.40 benchmark.

### Confirmatory factor analysis

The congeneric one-factor model estimated by maximum likelihood (listwise *N* = 121) yielded *χ*^2^ (77) = 102.5, *p* = 0.025; CFI = 0.904; TLI = 0.887; RMSEA = 0.058. Overall model fit was acceptable, although only marginally so. Given the modest sample size and the three-category response format, these results were considered satisfactory for an initial validation study. Nevertheless, replication using a categorical estimator such as WLSMV is warranted (18, 32).

### Item response theory: graded response model

GRM discrimination parameters ranged from 0.77 to 1.75 (mean 1.26), spanning low to high discrimination, with most items in the moderate range (33) (Table 6). First threshold parameters were strongly negative for all items (−2.94 to −1.07), suggesting that the lowest response category did not present comprehension or endorsement difficulties. Second threshold parameters were mildly positive (0.05 to 1.06), indicating that the transition from “average” to “good” occurred slightly above the trait mean. Total test information peaked at approximately 7.1 slightly above the trait mean, with a minimum standard error close to 0.37. This corresponds to a marginal reliability of roughly 0.86 in that region (Figure 5).

**Table 6:** Item parameters of Samejima’s graded response model, estimated by marginal maximum likelihood: discrimination (a) and the two category thresholds on the latent trait metric (b₁ separates category 1 from categories 2–3; b₂ separates categories 1–2 from category 3).

Item	a	b₁	b₂
Streets and movement	1.21	−1.94	0.87
Natural spaces	1.10	−2.16	0.33
Places for play	1.73	−1.73	0.44
Walking and cycling	1.59	−1.72	0.19
Public transport	1.18	−1.45	0.69
Traffic and parking	1.58	−1.07	0.62
Feeling heard	0.77	−1.81	1.06
Care and cleanliness	1.72	−1.07	0.76
Feeling safe	1.75	−1.57	0.32
Identity and pride	0.88	−2.94	0.05
Services and amenities	0.86	−2.59	0.22
Work and economy	1.14	−2.70	0.60
Housing and neighbours	0.89	−2.92	0.16
Social contact	1.21	−2.12	0.53

**Figure 5:**
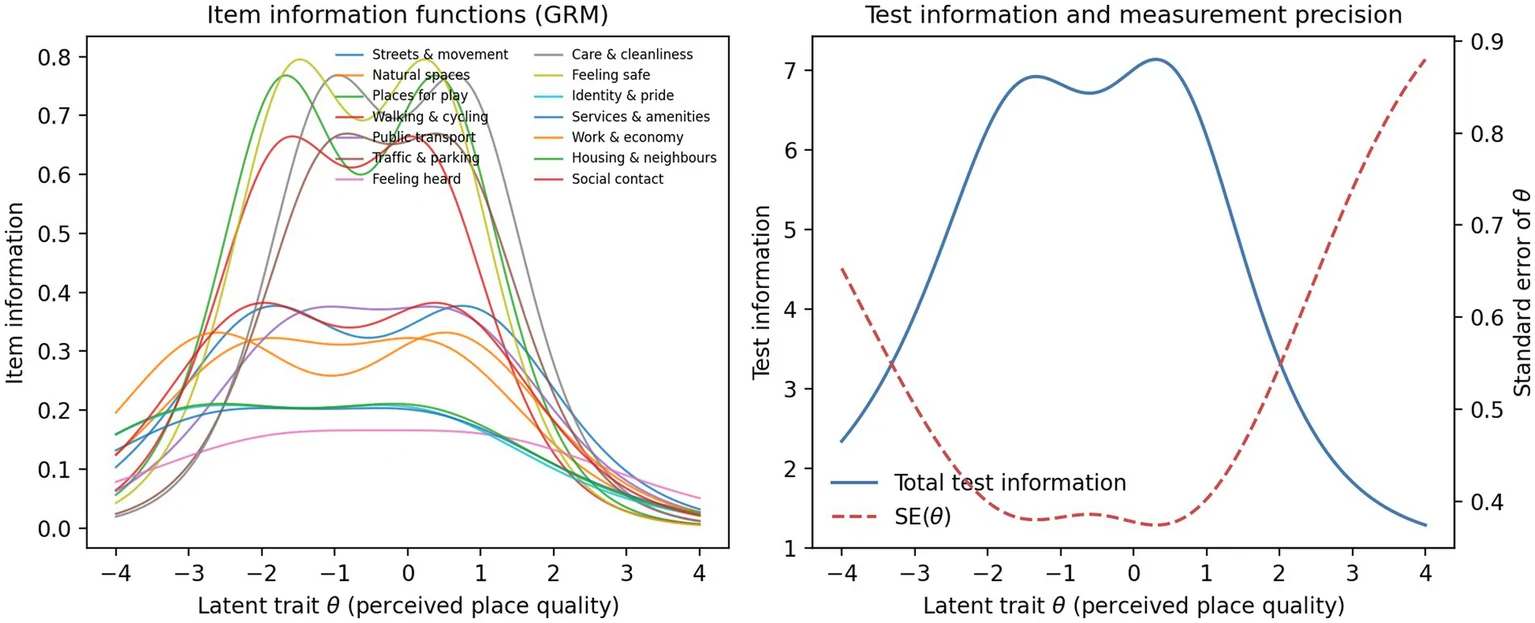
Measurement precision of the PST-C under the graded response model. Left: Item information functions. Right: Total test information, including the contribution of the standard normal prior, and the corresponding standard error of the latent trait.

### Known-groups validity

After Holm correction for multiple testing across the 14 comparisons, no statistically significant differences were observed by sex (Mann–Whitney U), school or grade level (Kruskal-Wallis). Possible interpretations of these null findings are considered in the Discussion.

### Cross-language semantic equivalence

Cosine similarities between the English source items and their Italian translations averaged 0.86 (range 0.65 to 0.94; Table 7, Figure 6). The two open-ended prompts achieved similarity scores of 0.86 and 0.90, respectively. Matched item pairs were approximately two to three times more similar than the benchmark similarities obtained from non-matching items (0.31 to 0.38), indicating that each Italian item was semantically much closer to its corresponding English source item than to any other domain. The lowest value was observed for *Feeling heard* (0.65), whose English wording is the most idiomatic and conceptually abstract among the fourteen domains.

**Table 7:** Cross-language semantic equivalence of the Italian PST-C, computed with the paraphrase-multilingual-MiniLM-L12-v2 sentence-embedding model.

Item	Cosine similarity, matched pair	Mean similarity, non-matching items
Streets and movement	0.91	0.32
Natural spaces	0.91	0.36
Places for play	0.85	0.37
Walking and cycling	0.92	0.32
Public transport	0.78	0.31
Traffic and parking	0.89	0.36
Feeling heard	0.65	0.32
Care and cleanliness	0.82	0.36
Feeling safe	0.90	0.35
Identity and pride	0.94	0.32
Services and amenities	0.91	0.31
Work and economy	0.86	0.38
Housing and neighbours	0.91	0.34
Social contact	0.82	0.35
Open prompt: “What is good now?”	0.86	n/a
Open prompt: “What could be improved?”	0.90	n/a

For each item: Cosine similarity between the English source and its Italian translation (matched pair), and mean similarity between the Italian item and the English source items of the other 13 domains (benchmark). The last two rows refer to the open-ended prompts, for which no benchmark applies.

**Figure 6:**
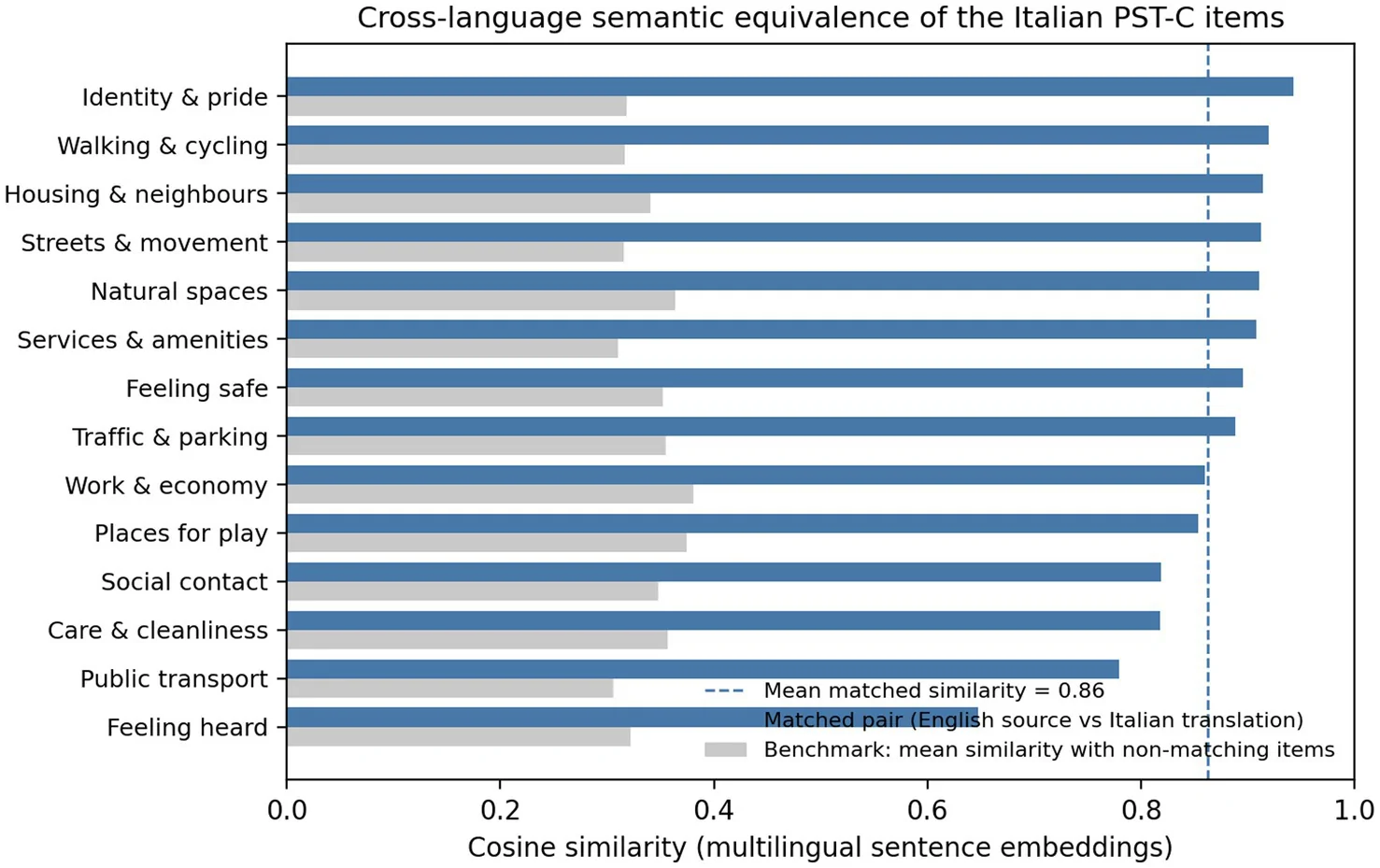
Semantic equivalence between the English source and the Italian translation of each PST-C item (blue bars), compared with the mean similarity between each Italian item and the source items of the other domains (grey bars), based on multilingual sentence embeddings. Items are sorted by matched-pair similarity; the dashed line marks the mean (0.86).

## Discussion

To our knowledge, this is one of the first validation studies of a child-centered environmental assessment instrument to integrate COSMIN standards, item response theory (Samejima’s graded response model), and AI-based semantic evaluation using multilingual sentence embeddings. This integrated framework extends conventional validation procedures by combining psychometric evaluation with objective assessment of cross-language semantic equivalence. The present study was conducted within the PNC Clima programme, which seeks to investigate the relationships between environment, climate, and health from a One Health perspective. In this context, the availability of a validated Italian PST-C is particularly relevant because it enables the systematic inclusion of children’s perceptions within broader environmental-health monitoring activities.

The instrument demonstrated an essentially unidimensional structure. Parallel analysis, scree plot inspection, exploratory factor analysis, and confirmatory factor analysis all supported the presence of a single general factor of perceived place quality underlying the fourteen theoretical domains. Moreover, all polychoric factor loadings were positive and substantively meaningful. Although the single factor explained approximately one-third of the total variance, the convergence of evidence across multiple psychometric approaches supports the interpretation of the PST-C as measuring a dominant latent construct. Internal consistency was good (*α* = 0.83; *ω* = 0.86), with bootstrap confidence intervals remaining above the conventional 0.70 threshold, indicating that the total scores are sufficiently reliable for applied participatory research and practice. The modest advantage of ω over α is expected, given that ω does not assume tau-equivalence among items (26, 27). The embedding-based analysis provides an additional source of evidence that cannot be obtained through qualitative translation procedures alone: the Italian items showed high semantic similarity to their English source versions, and the one domain displaying the greatest divergence (*Feeling heard*) was clearly identified as a potential target for future refinements. These reliability estimates are comparable to those reported for adult PST adaptations, including the Cronbach’s α of 0.89 reported for the Macedonian version (14).

The item response theory findings further refine this interpretation. Measurement precision was highest slightly above the mean of the latent trait, where the instrument most effectively distinguishes children who rate their living environment as “average” from those who rate it as “good.” The consistently negative first thresholds suggest that the lowest response category contributes relatively little information across the trait continuum. Four items (*Feeling heard, Identity and pride, Services and amenities* and *Housing and neighbours*) emerged as comparatively weaker indicators, showing corrected item-total correlations below 0.40, GRM discrimination parameters below 1.0 and polychoric loadings below 0.50. Notably, these items represent the more affective, relational, or interpretive dimensions of place experience, including feeling heard, pride, access to services and neighborhood relations. By contrast, the strongest items were associated with more concrete environmental characteristics, such as opportunities for play, traffic conditions, orderliness and safety. Their removal would not meaningfully improve reliability, and would reduce the conceptual breadth of the construct being measured.

Beyond its psychometric properties, the validated Italian PST-C has potential relevance for environmental health research and participatory urban governance. Contemporary approaches to child-friendly cities, climate adaptation and One Health increasingly recognize that children’s subjective experiences of neighbourhood environments complement objective indicators such as green space availability, walkability or environmental exposures. The PST-C provides a structured mechanism through which children’s perspectives can be systematically collected and incorporated into planning and evaluation processes. It complements the other ways in which children’s experience of place can be documented rather than replacing them. What it adds is comparability: ratings that can be set side by side across children, classes and neighbourhoods. Used on its own, though, it cannot capture the relational, affective and contextual dimensions of how children inhabit the places they use every day, and we treat it as one component of a broader participatory repertoire rather than a stand-alone assessment. Within the PNC Clima programme, the instrument may support future efforts to integrate perceptions of place quality with environmental, climatic and health data, contributing to a more comprehensive understanding of how children experience and respond to their everyday environments.

## Limitations

Several limitations should be acknowledged. First, ceiling effects were observed for all items, with four items exceeding 40%. This likely reflects the restricted three-point response scale, the generally positive response tendencies of younger children and the relatively homogeneous environmental characteristics of the participating schools, all of which are located within the same municipality. Future studies should evaluate whether a five-point response scale with clearly defined verbal anchors improves score dispersion while preserving comprehensibility.

Second, known-groups validity was not supported after correction for multiple testing. Although this may reflect genuine similarities in perceived place quality across sex, schools, and grade levels, the modest sample size, limited environmental heterogeneity, and conservative multiplicity correction are more likely to have reduced statistical power. Larger and more diverse samples are therefore needed to further evaluate this measurement property.

Third, the confirmatory model was estimated using maximum likelihood despite the ordinal nature of the data. Although model fit was acceptable, future studies should replicate the analyses using weighted least squares with mean and variance adjustment (WLSMV), the preferred estimator for ordinal items (18, 32). Fourth, the sample came from a single urban context and is not nationally representative. The single-city sampling frame (four state comprehensive institutes in Palermo) and the item-level missing data (up to 9.2%), part of which follows from children exercising their right to skip questions or to stop, limit how far the findings generalize. Multi-site studies covering northern, central and southern Italy, and both urban and rural settings, are needed before the Italian PST-C can be recommended for national use.

A last limitation is conceptual rather than statistical. The PST-C gives a structured, comparable and quickly administered picture of how children rate their environment, but a rating on a three-point scale is a compressed record of a complex experience. It can tell us that a child scores traffic poorly. It cannot tell us which street, at which time of day, or what it feels like to cross it. Qualitative and participatory methods reach that layer of meaning better: individual and group interviews, focus groups, and visual methods such as photo elicitation, child-led walking tours, drawing and mapping activities, all of which let children take a more active part in producing and interpreting knowledge about the places they use every day (35, 36). We see the PST-C and these approaches as complementary rather than alternative. Mixed-methods designs, in which PST-C scores identify the domains that matter most in a given place and qualitative work then asks why, seem to us the direction worth pursuing with the Italian version.

## Conclusion

The Italian PST-C provided a reliable measure of perceived place quality among primary school children, with evidence supporting a clear unidimensional structure and overall high semantic equivalence to the English version. The instrument appears suitable for the participatory assessment of children’s living environments in Italian schools, both within the Italian PNC Clima programme and in a broader research and planning context. However, interpretation of total scores should take into account the ceiling effects associated with the current three-point response format.

## Data Availability

The raw data supporting the conclusions of this article will be made available by the authors, without undue reservation.
